# Neutrophils in the era of single-cell RNA sequencing: functions and targeted therapies in cancer

**DOI:** 10.20892/j.issn.2095-3941.2024.0012

**Published:** 2024-02-05

**Authors:** Jing Qin, Feng Wei, Xiubao Ren

**Affiliations:** 1 National Clinical Research Center for Cancer, Tianjin Medical University Cancer Institute & Hospital, Tianjin 300060, China; 2 Tianjin’s Clinical Research Center for Cancer, Tianjin 300060, China; 3 Key Laboratory of Cancer Prevention and Therapy, Tianjin 300060, China; 4 Key Laboratory of Cancer Immunology and Biotherapy, Tianjin 300060, China; 5 Department of Immunology, Tianjin Medical University Cancer Institute & Hospital, Tianjin 300060, China; 6 Department of Biotherapy, Tianjin Medical University Cancer Institute & Hospital, Tianjin 300060, China

## Introduction

Neutrophils, a crucial component of the innate immune system, are the most abundant bone marrow-derived eukaryotic cell in human blood. The functional importance of neutrophils is often overlooked because neutrophils are short-lived, terminally differentiated, and do not proliferate. Although traditionally considered anti-bacterial cells, research has demonstrated the multifaceted roles of neutrophils in cancer. Studies have suggested that neutrophils with normal functions co-exist with pathologically activated neutrophils that support tumor progression and metastasis in the context of cancer. Based on fluorescence-activated cell sorting (FACS) technology, human peripheral blood CD66b+ high-density neutrophils (HDNs) have been designated classical neutrophils with normal functions and CD66b+ low-density neutrophils (LDNs) have been designated polymorphonuclear myeloid-derived suppressor cells (PMN-MDSCs) or tumor-associated neutrophils (TANs) with protumor functions^1^. However, in many studies TANs are specifically referred to as neutrophils that infiltrate tumor tissues. HDNs are tumor-cell lethal and are commonly referred to as N1 TANs, whereas LDNs are usually associated with impaired functions and immunosuppressive properties and are N2 TANs. Owing to the confusing terminology, neutrophils in tumor tissues are referred to as TANs in the current study. PMN-MDSCs were shown to be like N2 TANs with shared protumor properties in one study^2^.

In recent years researchers have identified TANs with various anti-tumor functions, including direct cytotoxicity against tumor cells and inhibition of metastasis. However, it was also shown that TANs support tumor progression by promoting angiogenesis, stimulating tumor cell motility, and acting as an immunosuppressive switch to regulate the behavior of other immune cells. Thus, TANs are increasingly recognized as important regulators of cancer progression. Evidence from experimental tumor models and cancer patients has demonstrated the significant morphologic and functional heterogeneity of TANs and dual pro- and anti-tumor functions of TANs in cancer. Thus, understanding how to enhance anti-tumor functions while suppressing pro-tumor functions is a major challenge with high significance for cancer treatment. In addition, the development of single-cell RNA sequencing (scRNA-seq) technology has led to breakthroughs in our understanding of neutrophil heterogeneity. Herein we meticulously describe the current research findings on neutrophil properties, how these properties can be used for targeted treatment, and how scRNA-seq technology has enabled us to further explore potential therapeutic targets.

## Pro-tumor functions of neutrophils

Regarding the pro-tumor effects of neutrophils, neutrophil extracellular traps (NETs), which are net-like structures consisting of DNA fibers, histones, and proteins, must be highlighted as a key point^3^. Within the tumor area, NETs contribute to distant metastasis by disrupting the normal connections between endothelial cells, which allows tumor cells to extravasate and complete implantation metastasis. NETs may block the cytotoxic abilities of CD8+ T and natural killer (NK) cells against tumor cells^4^. Within the pre-metastasis niche, NETs have been shown to attract and capture cancer cells to form distant metastases in mouse models^5^. Circulating tumor cells (CTCs) encapsulated by NETs can form new metastases. A recent study showed that NETs promote tumor progression by the metabolic reprogramming of naïve CD4+ T cells to differentiate into Treg cells, which suppress cancer immune surveillance^6^.

**Figure 1:**
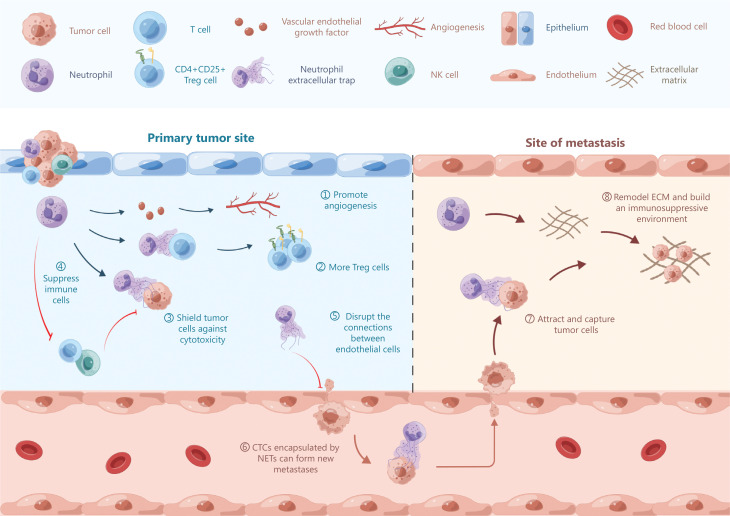
Pro-tumor functions of neutrophils. The pro-tumor functions of neutrophils can be divided into three categories depending on the sites: primary tumor site (①∼⑤); circulation (⑥); and site of metastasis (⑦, ⑧). NETs, neutrophil extracellular traps; CTCs, circulating tumor cells; ECM, extracellular matrix.

In addition to NETs, the neutrophil pro-tumor functions are also reflected in other ways. First, neutrophils promote angiogenesis and lymphangiogenesis in solid tumors and contribute to tumor growth and metastasis by releasing high levels of matrix metallopeptidase-9 (MMP9) and vascular endothelial growth factor (VEGF)^7^. A previous study provided evidence that neutrophils represent a major source of VEGF expression in the non-small cell lung cancer (NSCLC) tumor microenvironment (TME)^8^. Second, neutrophils directly suppress the anti-tumor functions of immune cells and promote tumor growth. For example, the nitric oxide produced by neutrophils has been shown to kill effector tumor-specific T cells^9^. Neutrophil-derived H_2_O_2_ inhibits NK cell function, which decreases tumor clearance and promotes lung metastasis in mouse models^10^. By enhancing the FAS signaling pathway and inhibiting IL-8 secretion, neutrophils inhibit NK cell activation and even cause NK cell apoptosis. Finally, TANs facilitate the survival and seeding of metastatic cells by remodeling the extracellular matrix (ECM) and building an immunosuppressive environment^11^ (**Figure 1**).

## Anti-tumor functions of neutrophils

NETs have well-known pro-tumor functions, but paradoxically NETs also exhibit anti-tumor activities. The myeloperoxidase (MPO) and proteases in NETs can kill tumors and inhibit tumor growth and metastasis. Indeed, NETs are positively correlated with the prognosis of patients with head and neck squamous cell carcinoma^12^. An *in vitro* experiment reported that the migration and viability of melanoma cells are decreased when co-incubated with NETs, suggesting cytotoxic effects of NETs^13^.

Like NETs, neutrophil-derived reactive oxygen species (ROS) and reactive nitrogen species (RNS) have anti-tumor functions. The H_2_O_2_ produced by neutrophils induce tumor cell death by increasing the Ca^2+^ concentration in tumor cells to lethal levels *via* transient receptor potential melastatin-related 2, an H_2_O_2_-dependent Ca^2+^ channel^14^. Neutrophil-produced NO also mediates tumor cell death^15^. Balancing the roles of NETs, ROS, and RNS secreted by TANs in the TME and inhibiting pro-tumor activity while exerting anti-tumor activity will be the focus of further studies.

**Figure 2:**
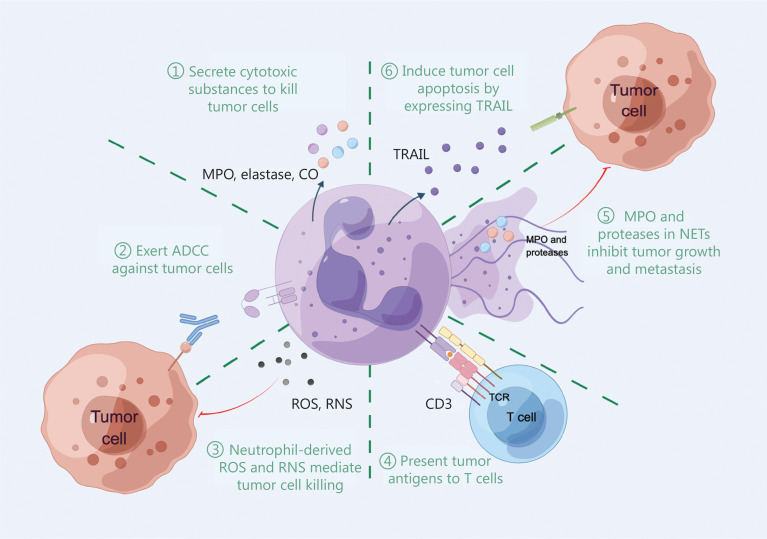
Anti-tumor functions of neutrophils. The anti-tumor functions of neutrophils can be demonstrated in six ways: direct cytotoxicity to tumor cells (①, ②, ③); tumor antigen presentation (④); anti-tumor effects of NETs (⑤); and induction of apoptosis in tumor cells (⑥). MPO, myeloperoxidase; CO, carbon monoxide; ADCC, antibody-dependent cell-mediated cytotoxicity; ROS, reactive oxygen species; RNS, reactive nitrogen species; TCR, T cell receptor; NETs, neutrophil extracellular traps; TRAIL, tumor necrosis factor-related apoptosis-inducing ligand.

In addition to these mechanisms, neutrophils execute anti-tumor functions through numerous pathways. Decades ago it was demonstrated that neutrophils induce antibody-dependent cell-mediated cytotoxicity (ADCC) against solid tumor cells. Moreover, neutrophils secrete cytotoxic substances, such as MPO, elastase, and carbon monoxide, which exert substantial tumor cell cytotoxicity. TANs induce tumor cell apoptosis by expressing the tumor necrosis factor-related apoptosis-inducing ligand (TRAIL)^16^. Finally, studies have confirmed the existence of a special subset of TANs capable of cross-presenting exogenous tumor antigens to CD8+ T cells, which in turn stimulate tumor-specific effector T cell responses^8^ (**Figure 2**).

## Neutrophil-dependent therapies

**Figure 3:**
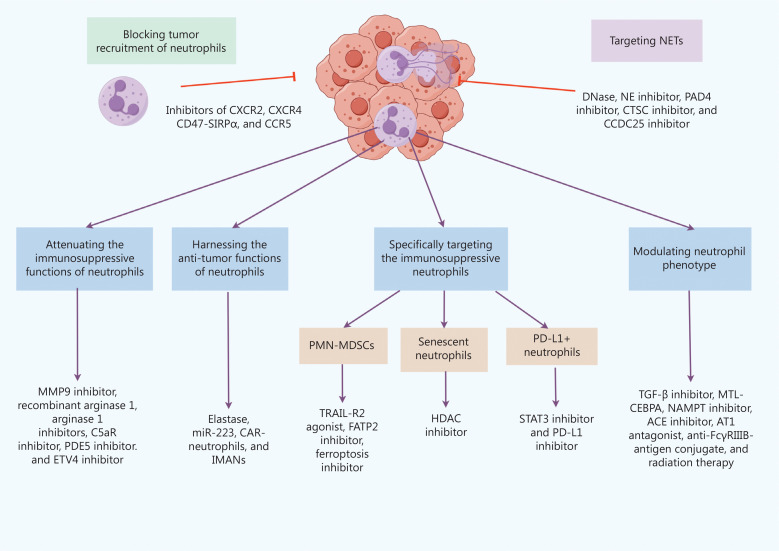
Therapies dependent on neutrophils. Neutrophil-dependent therapies are mainly divided into six categories: blocking tumor recruitment of neutrophils; attenuating the immunosuppressive functions of neutrophils; harnessing the anti-tumor functions of neutrophils; specifically targeting the immunosuppressive neutrophils; modulating neutrophil phenotype; and targeting NETs. CXCR2, C–X–C chemokine receptor 2; CXCR4, C–X–C chemokine receptor 4; CCR5, C–C chemokine receptor 5; MMP9, matrix metalloproteinase 9; C5aR, C5a receptor; PDE5, phosphodiesterase type 5; CAR-neutrophils, chimeric antigen receptor-neutrophils; IMANs, immunomodulatory alpha neutrophils; TRAIL-R2, tumor necrosis factor-related apoptosis-inducing ligand receptor 2; FATP2, fatty acid transport protein 2; HDAC, histone deacetylase; STAT3, signal transducer and activator of transcription 3; TGF-β, transforming growth factor-β; NAMPT, nicotinamide phosphoribosyltransferase; ACE, angiotensin converting enzyme; AT1, angiotensin II type I; DNase, deoxyribonuclease; NE, neutrophil elastase; PAD4, peptidylarginine deiminase 4; CTSC, cathepsin C.

Based on the heterogeneity of neutrophil functions, current therapeutic strategies targeting neutrophils include the following six options (**Figure 3**):

1. Blocking tumor recruitment of neutrophils
  Targeting neutrophils is challenging owing to the lack of tractable targets; however, inhibition of C–X–C chemokine receptor 2 (CXCR2) and its ligand, C–X–C chemokine ligand 8 (CXCL8, also known as IL-8), has been used to prevent neutrophil recruitment to tissues^17^. A recent study suggested that the CXCR2 inhibitor, AZD5069, is a promising approach to improve triple-negative breast cancer treatment because the blockade of CXCR2 significantly reduces the infiltration of neutrophils and improves the efficacy of immune checkpoint blockade in *in vitro* tests^18^. Tumor cells also induce neutrophil extrusion of NETs by secreting CXCR2 ligands^4^. Agents targeting CXCR2 (AZD5069, reparixin, navarixin, and SX-682) and CXCL2 [BMS-986253 (also known as HuMax-IL8)] are currently being investigated for anti-tumor effects in ongoing phase I/II clinical studies in combination with chemotherapy and immune checkpoint inhibitors (ICIs) in several tumor types; the results warrant further follow-up (**Table 1**).
  Conventional CXCR2 inhibitors indiscriminately inhibit the infiltration of pro- and anti-tumor neutrophils into tumors; however, among tumors in which anti-tumor neutrophils are originally predominant, inhibition of neutrophil recruitment is counterproductive. Moreover, CXCR2 regulates the release of bone marrow neutrophils. Inhibition of CXCR2 induces neutropenia, causes innate immune deficiency, and increases the risk of infection, which limits the maximum clinical dose and reduces efficacy.
  In addition to CXCR2 inhibitors, preclinical studies have also demonstrated that inhibitors of CXCR4, C–C chemokine receptor 5 (CCR5), and CD47-SIRPα pathway limit neutrophil migration to tumor areas; these strategies are being investigated in multiple clinical trials (**Table 1**).
2. Attenuating neutrophil immunosuppressive functions
  In addition to stimulating angiogenesis, a recent study reported that MMP9 activates the latent TGF-β captured in the NET to promote epithelial-to-mesenchymal (EMT) transition and chemotherapy resistance^19^. MMP9 is specifically expressed in neutrophils, suggesting that MMP9 could be an effective target. A phase III clinical trial (GAMMA-1) showed that compared to the mFOLFOX6 group, the addition of an MMP9 inhibitor, andecaliximab (ADX), led to an improvement in overall survival (OS) in patients > 65 years of age, but did not improve the OS in patients with gastric adenocarcinoma^20^. MMP9 inhibitors are promising anti-tumor agents; however, further investigations are required to assess the clinical potential.
  Other targets that trigger neutrophil immunosuppressive functions include arginase 1 inhibitor, recombinant arginase 1, C5a receptor (C5aR), and phosphodiesterase type 5 (PDE5), all of which have entered clinical trials (**Table 1**). A recent study showed that the transcription factor, ETV4, has been identified as a potential target with the capacity to inhibit TAN-mediated lymphangiogenesis and lymph node metastasis in a preclinical model^7^, which warrants further exploration.
3. Harnessing anti-tumor functions of neutrophils
  Recent studies have identified several potential targets for harnessing the anti-tumor functions of neutrophils. First, neutrophil elastase, a protease released by neutrophils, has a selective cytotoxic effect on cancer cells but spares host cells. Therefore, harnessing elastase potentially augments therapeutic effects while ensuring patient safety^21^. Second, miR-223 is highly expressed in neutrophils and can be delivered intracellularly *via* extracellular vesicles. By directly targeting HIF-1α in hepatocellular carcinoma cells, miR-223 was reported to significantly reverse hypoxia-driven immunosuppression and angiogenesis, thus slowing down tumor progression in mouse models^22^. Third, neutrophils effectively cross physiologic barriers, such as the blood-brain barrier in patients with glioblastoma (GBM). Using drug-loaded chimeric antigen receptor (CAR)-neutrophils as drug carriers, GBM cells were efficiently killed *in vitro* and the lifespan of tumor-bearing mice was prolonged^23^. Finally, immunomodulatory alpha neutrophils (IMANs), which are generated from hematopoietic stem cells of donors with exceptional anti-tumor innate immunity, exhibit potent cytotoxic potential in pancreatic ductal adenocarcinoma tumor models.
  It is worth noting that the abovementioned potential targets were identified in preclinical studies. Whether the targets can be utilized in clinical trials warrants further study.
4. Specifically targeting immunosuppressive neutrophils
  Several therapeutic strategies are currently available for targeting PMN-MDSCs. Prospective studies involving tumor necrosis factor-related apoptosis-inducing ligand receptor 2 (TRAIL-R2) agonists have shown selective reduction in PMN-MDSCs^24^. In addition, suppression of fatty acid transport protein 2 (FATP2) weakens the immunosuppressive effect of PMN-MDSCs and creates a new means to specifically target suppressive neutrophils by reducing prostaglandin E2 synthesis^25^. Moreover, ferroptosis inhibitor suppresses the immunosuppressive functions of PMN-MDSCs, thus inhibiting tumor progression in a mouse model^26^.
  In addition to PMN-MDSCs, it is possible to intervene with other immunosuppressive neutrophils, such as senescent and programmed cell death ligand-1 (PD-L1)-positive neutrophils. A recent study reported that histone deacetylase (HDAC) inhibition eliminates senescent neutrophils, which have increased pro-tumor activity than normal neutrophils, thus improving therapeutic efficacy^27^. Another study suggested that PD-L1^+^ neutrophils suppress T cell immunity *via* the signal transducer and activator of transcription 3 (STAT3)-PD-L1 pathway within the TME^28^. Therefore, targeting these pro-tumor neutrophils *via* STAT3 and PD-L1 inhibitors may provide novel treatment strategies.
  Several clinical trials involving TRAIL-R2 agonists, HDAC inhibitors, and STAT3 inhibitors are currently underway (**Table 1**). PD-L1 inhibitors are often combined with other drugs targeting neutrophils in clinical trials. FATP2 and ferroptosis inhibitors have been used in preclinical studies. Whether FATP2 and ferroptosis inhibitors can be utilized in clinical trials warrants further exploration.
5. Modulating neutrophil phenotypes
  The purpose of modulating neutrophil phenotypes is to convert pro-tumor N2 neutrophils into anti-tumor N1 neutrophils. First, studies have demonstrated that in an environment enriched with transforming growth factor (TGF)-β, neutrophils usually have an N2 phenotype, while in the presence of a TGF-β inhibitor, neutrophils shift to an N1 phenotype^29^. Second, the CCAAT/enhancer-binding protein (C/EBP) transcription factor has been suggested to be MDSC immunosuppressive activity-dependent^30^. MTL-CEBPA is an RNA-based agent that may convert pro-tumor neutrophils into anti-tumour neutrophils. Third, nicotinamide phosphoribosyltransferase (NAMPT) inhibitors could shift neutrophils to an anti-tumor phenotype^31^. Clinical trials involving TGF-β inhibitors, MTL- CEBPA, and NAMPT inhibitors are ongoing (**Table 1**).
  Several other potential targets have shown promising results in preclinical studies. Angiotensin-converting enzyme (ACE) inhibitors and angiotensin II type I (AT1) receptor antagonists can reprogram neutrophils towards an anti-tumor phenotype^32^. Second, FcγRIIIB is a receptor selectively expressed on neutrophils. An anti-FcγRIIIB-antigen conjugate is a promising therapeutic approach because neutrophils can be induced to adopt properties of dendritic cells and promote the anti-tumor immunity of CD8+ T cells^33^. Finally, another study showed that radiation therapy can also polarize immunosuppressive TANs into an anti-tumor phenotype^34^.
6. Targeting NETs
  Animal studies have shown that small molecule drugs, such as deoxyribonuclease (DNase), neutrophil elastase (NE) inhibitors, peptidylarginine deiminase 4 (PAD4) inhibitors, and cathepsin C (CTSC) inhibitors, block NET formation, which inhibits tumor metastasis. DNase I digests NETs and suppresses the growth of established liver micrometastases in animal models^35^. NE inhibitors and PAD4 inhibitors block NET formation by preventing chromatin decondensation^36^. Xiao et al.^37^ reported that the tumor-secreted protease, CTSC, induces the formation of NETs through the CTSC–PR3–IL-1β axis. Targeting CTSC with AZD7986 effectively suppresses circulating pulmonary NETs and alleviates lung metastasis of breast cancer in a mouse model^37^. Clinical trials on DNase are currently ongoing.
  In addition to blocking NET formation, a recent study showed that CCDC25, a transmembrane protein that functions as a NET-DNA receptor in cancer cells, holds promise as a target to mitigate NET-mediated cancer metastasis^5^. It is noteworthy, however, that CCDC25 has near-indiscriminate expression in human tissues. Therefore, systematic inhibition of CCDC25 may be risky and should be carefully considered when designing clinical studies.

**Table 1:** Clinical trials of drugs targeting neutrophils

Drug	Phase	Disease	Trial identifier	Study status
CXCR2/IL-8 antagonists								
AZD5069	I/II	Squamous cell carcinoma of head and neck	NCT02499328	Active, not recruiting
	I/II	PDAC	NCT02583477	Completed
	I/II	Metastatic castrate-resistant prostate cancer	NCT03177187	Terminated
BMS-986253	I/II	Hormone-sensitive prostate cancer	NCT03689699	Active, not recruiting
	I/II	HCC	NCT04050462	Active, not recruiting
	I	HCC, NSCLC	NCT04123379	Recruiting
	I/II	Metastatic or unresectable solid tumours	NCT03400332	Recruiting
	I	Metastatic solid tumours	NCT04572451	Recruiting
SX-682	I	Metastatic melanoma	NCT03161431	Recruiting
	I	PDAC	NCT04477343	Recruiting
	I	Myelodysplastic syndrome	NCT04245397	Recruiting
Navarixin/MK-7123	II	Advanced/metastatic solid tumors	NCT03473925	Completed
Reparixin	II	Breast cancer	NCT02370238	Completed
	I	Breast cancer	NCT02001974	Completed
C/EBPα								
MTL-CEBPA small activating RNA	I	HCC	NCT02716012	Unknown status
	I	Solid tumors	NCT04105335	Active, not recruiting
TGF-β pathway inhibitors								
Galunisertib	II	Glioblastoma	NCT01582269	Active, not recruiting
	I	Glioma	NCT01682187	Active, not recruiting
	I	Ovarian carcinosarcoma	NCT03206177	Unknown status
	I	Breast cancer	NCT02672475	Active, not recruiting
	II	Metastatic castration-resistant prostate cancer	NCT02452008	Recruiting
	II	Rectal cancer	NCT02688712	Active, not recruiting
	II	Nasopharyngeal carcinoma	NCT04605562	Not yet recruiting
	I	HCC	NCT02240433	Completed
	II	HCC	NCT01246986	Completed
	I/II	HCC, Solid tumors and NSCLC	NCT02423343	Completed
	I	PDAC	NCT02734160	Completed
Fresolimumab (GC1008)	I/II	NSCLC	NCT02581787	Completed
IMC-TR1	I	Neoplasms	NCT01646203	Completed
STAT3 inhibitors								
Napabucasin	III	Colorectal cancer	NCT02753127	Completed
	I	HCC	NCT02358395	Completed
	I/II	HCC	NCT02279719	Completed
TTI101	I	HCC	NCT03195699	Active, not recruiting
Danvatirsen	I	HCC	NCT01839604	Completed
Lcaritin	I	HCC	NCT02496949	Completed
	II	HCC	NCT01972672	Completed
	III	HCC	NCT03236636	Unknown status
	III	HCC	NCT03236649	Terminated
TRAIL receptor 2 agonists								
Tigatuzumab	II	Breast cancer	NCT01307891	Completed
	II	HCC	NCT01033240	Completed
CS-1008	I	Colorectal neoplasms	NCT01220999	Completed
DS8273a	I	Solid tumors and lymphoma	NCT02076451	Completed
	I	Colorectal cancer	NCT02991196	Terminated
	I	Melanoma	NCT02983006	Completed
CD47-SIRPα pathway inhibitors								
Hu5F9-G4	I	Solid tumors	NCT02216409	Completed
IBI188	I	Advanced malignancies	NCT03717103	Completed
CC-90002	I	Hematologic neoplasms	NCT02367196	Completed
Magrolimab	I/II	Colorectal cancer	NCT02953782	Completed
TTI621	I	Solid tumors	NCT02890368	Terminated
AO176	I	Solid tumors	NCT03834948	Completed
BI765063	I	Solid tumors	NCT03990233	Active, not recruiting
Recombinant arginase 1/arginase 1 inhibitors								
Pegzilarginase	I/II	Extensive disease small cell lung cancer	NCT03371979	Completed
INCB001158	I/II	Advanced/Metastatic solid tumors	NCT02903914	Completed
	I/II	Solid tumors	NCT03314935	Completed
Anti-C5aR								
IPH5401	I	Advanced solid tumors	NCT03665129	Terminated
PDE5 inhibitors								
Tadalafil	I	Liver dominant colorectal cancer or PDAC	NCT03785210	Completed
	II	Head and neck cancers	NCT02544880	Completed
Sildenafil	II/ III	NSCLC	NCT00752115	Completed
HDAC inhibitors								
Resminostat	II	HCC	NCT00943449	Completed
Belinostat	I/II	HCC	NCT00321594	Completed
CCR5 inhibitor								
Maraviroc	I	Colorectal cancer	NCT03274804	Completed
	I	Colorectal cancer	NCT01736813	Completed
CXCR4 inhibitor								
Plerixafor	I	PDAC, colorectal and ovarian cancer	NCT02179970	Completed
NAMPT inhibitor								
Daporinad	II	Cutaneous T cell lymphoma	NCT00431912	Completed
MMP9 inhibitor								
Andecaliximab	III	Gastric adenocarcinoma	NCT02545504	Completed
NETs inhibitors								
Pulmozyme (rhDNase)	I	Head and neck cancers	NCT00536952	Unknown status
Oshadi D	II	Acute myeloid/lymphoid leukemia	NCT02462265	Suspended

PDAC, pancreatic ductal adenocarcinoma; HCC, hepatocellular carcinoma; NSCLC, non-small cell lung cancer.

## Exploring neutrophil heterogeneity using scRNA-seq

Recently, scRNA-seq technology has become a new tool for elucidating the mechanisms underlying neutrophil functions; however, uncovering the true nature of neutrophils can be arduous. First, because neutrophils are fragile *in vitro*, significant loss during sample preparation makes it difficult to capture neutrophils. Second, the RNA content of neutrophils is lower than other cell types, which poses a technologic challenge. Third, proteases in neutrophil granules may disrupt the cell capture process during scRNA-seq. Consequently, in human studies neutrophils are generally missing or severely underestimated compared to the expected proportion, which greatly limits determination of neutrophil heterogeneity. However, two feasible methods for solving these problems have been identified: 1) BD Rhapsody (BD Biosciences), a suitable scRNA-seq platform, captures significantly more mRNA molecules per cell than 10× Chromium (10× Genomics) and is better for studying neutrophils^8^. 2) An alternative method of data analysis has been devised which significantly increases neutrophil capture.

The scRNA-seq technology has provided unprecedented insight into the complexity of neutrophil subpopulations. The simple classification of neutrophils into N1 and N2 TANs is considered an oversimplification because studies have suggested that neutrophils display a range of functional phenotypes. With the progressive understanding of neutrophil heterogeneity, it has become an important issue to determine whether regulation of specific subpopulations reduces pro-tumor effects and maximizes anti-tumor effects.

A subpopulation with high expression of IFN-stimulated genes (ISG) was recently identified through scRNA-seq of peripheral blood neutrophils from healthy populations, representing a specific cellular state^38^. These neutrophils with high ISG expression are likely to be tissue-damaging, immunostimulatory, and exist in small amounts in healthy populations. Under pathologic conditions, such as patients with systemic lupus erythematosus, scRNA-seq has demonstrated that neutrophil type 1 IFN signaling is overactive. A recent study suggested that neutrophils with high ISG expression may represent an anti-tumor subpopulation^39^. It has been shown in mouse models that an expanded subpopulation of ISG-expressing neutrophils was positively associated with immunotherapy efficacy and that interferon-responsive transcription factor 1 (IRF1) may be a pivotal upstream modulator in generating these cells, thus serving as a therapeutic target. It has also been shown that lung cancer patients with an elevated neutrophil-to-lymphocyte ratio (NLR) after immunotherapy have significantly longer progression-free survival than patients with a decreased NLR, suggesting that an immunotherapy-induced systematic neutrophil response is positively associated with clinical benefits. In addition, one study identified a population of anti-tumor neutrophils with the ability to eliminate tumor antigen escape variants during T cell immunotherapy. Pseudotime analysis showed that upregulation of ISG expression was the differentiation direction of this special subpopulation, further demonstrating that high ISG expression characterizes the anti-tumor phenotype^40^.

A special pro-tumor neutrophil subpopulation with high expression of vascular endothelial growth factor A (VEGFA), lactate dehydrogenase A (LDHA), and basic helix-loop-helix transcription factor 40 (BHLHE40) was identified in human pancreatic ductal adenocarcinoma tumor tissues and was associated with a poor prognosis^41^. This study identified the transcription factor, BHLHE40, as a key regulator to drive neutrophils to a pro-tumor phenotype, suggesting the value of BHLHE40 as a therapeutic target. Another scRNA-seq analysis of hepatocellular carcinoma patients revealed that three pro-tumor neutrophil subpopulations [high expression of C-C chemokine ligand 4 (CCL4), phosphoprotein 1 (SPP1), and PD-L1] are associated with poor prognosis and that these neutrophils had the highest BHLHE40 activity, further confirming the feasibility of BHLHE40 as a therapeutic target^42^. In addition, both studies identified high ISG-expressing subpopulations. Although high ISG-expressing neutrophil function was not further investigated, it was suggested that ISG-expressing neutrophils are conserved in different tumor types.

**Figure 4:**
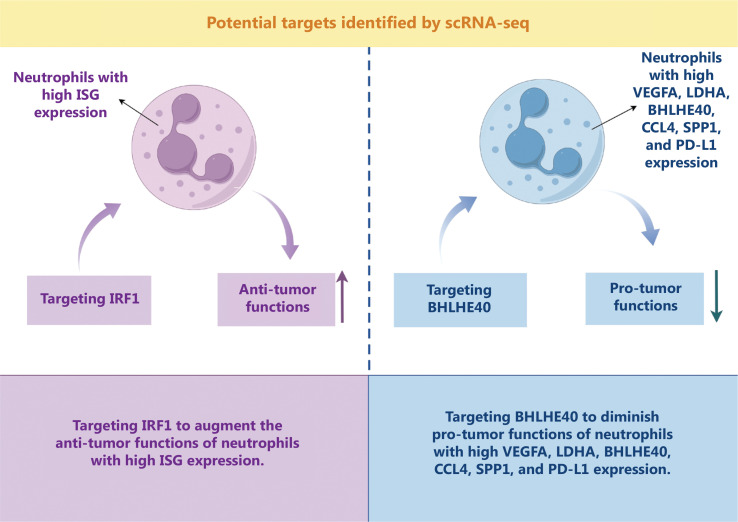
Potential targets of neutrophils identified by single-cell RNA sequencing (scRNA-seq). There are two main potential strategies: Enhancing anti-tumor functions of neutrophils with high ISG expression by targeting IRF1 and Attenuating pro-tumor functions of neutrophils with high VEGFA, LDHA, BHLHE40, CCL4, SPP1, and PD-L1 expression by targeting BHLHE40. ISG, IFN-stimulated genes; IRF1, interferon-responsive transcription factor 1; VEGFA, vascular endothelial growth factor A; LDHA, lactate dehydrogenase A; BHLHE40, basic helix-loop-helix transcription factor 40; CCL4, C-C chemokine ligand 4; SPP1, phosphoprotein 1; PD-L1, programmed cell death ligand-1.

It has been reported that the structures of neutrophil subpopulations are similar in different tumor types^39^, which was also the conclusion in the above studies. Pro- and anti-tumor neutrophils co-exist and may have different origins^39^. Collectively, IRF1 may be an effective target for augmenting the anti-tumor functions of neutrophils with high ISG expression, whereas the pro-tumor effects of neutrophils highly expressing VEGFA, LDHA, BHLHE40, CCL4, SPP1, and PD-L1 may be diminished by targeting BHLHE40 (**Figure 4**).

## Conclusions

Neutrophils have a complex role in cancer and are associated with tumor generation, progression, and metastasis. In addition to acting directly on tumor cells, neutrophils also have an indirect role by affecting angiogenesis and lymphangiogenesis, regulating immune cell effector function, altering the state of endothelial cell junctions, and remodeling the extracellular matrix to exert pro- or anti-tumor functions. Neutrophil targeting is a novel therapeutic approach for patients with cancer. Neutrophil-based therapeutic strategies can be broadly categorized into six groups: blocking the tumor recruitment of neutrophils; attenuating the immunosuppressive functions of neutrophils; harnessing the anti-tumor functions of neutrophils; specifically targeting immunosuppressive neutrophils; modulating neutrophil phenotypes; and targeting NETs. The scRNA-seq technique has enabled us to make a finer delineation of cell subpopulations, depending on which we can generate more specific therapeutic strategies and find possible targets in a high-resolution manner. The next critical step is to characterize these complex cell subpopulations at the functional level and to develop more reliable and specific therapeutic modalities by targeting pathologically active, suppressive neutrophils, while sparing classically active neutrophils with anti-tumor functions.
